# Vitamin D and Age-Related Macular Degeneration

**DOI:** 10.3390/nu9101120

**Published:** 2017-10-13

**Authors:** Alfredo Garcia Layana, Angelo Maria Minnella, Gerhard Garhöfer, Tariq Aslam, Frank G. Holz, Anita Leys, Rufino Silva, Cécile Delcourt, Eric Souied, Johanna M. Seddon

**Affiliations:** 1 Clínica Universidad de Navarra, University of Navarra, 31009 Pamplona, Spain; 2 Dipartimento di Scienze Otorinolaringoiatriche e Oftalmologiche, Universita’ Cattolica del Sacro Cuore, Lgo F. Vito 1, 00168 Roma, Italy; aminnella59@gmail.com; 3 Department of Clinical Pharmacology, University of Vienna, 1090 Vienna, Austria; gerhard.garhoefer@meduniwien.ac.at; 4 School of Pharmacy and Optometry, Faculty of Biology, Medicine and Health, University of Manchester, Manchester M13 9PL, UK; tariq.aslam@manchester.ac.uk; 5 Central Manchester University Hospitals NHS Foundation Trust, Manchester Academic Health Science Centre, M13 9WL Manchester, and Heriot Watt University, Edinburgh EH14 4AS, UK; 6 Department of Ophthalmology, University of Bonn, D-53107 Bonn, Germany; Frank.Holz@ukb.uni-bonn.de; 7 Department of Ophthalmology, University Hospitals Leuven, 3000 Leuven, Belgium; anita.leys@uzleuven.be; 8 Faculty of Medicine, Institute for Biomedical Imaging and Life Sciences (IBILI), University of Coimbra, 3000-548 Coimbra, Portugal; rufino.silva@oftalmologia.co.pt; 9 Centro Hospitalar e Universitário de Coimbra (CHUC), Department of Ophthalmology, 3000-548 Coimbra, Portugal; 10 Centro Hospitalar e Universitário de Coimbra (CHUC), Faculty of Medicine, Institute for Biomedical Imaging and Life Sciences (IBILI-FMUC), University of Coimbra, 3000-548 Coimbra, Portugal; 11 Centro Hospitalar e Universitário de Coimbra (CHUC), Association for Innovation and Biomedical Research on Light and Image (AIBILI), 3000-548 Coimbra, Portugal; 12 University of Bordeaux, INSERM, Bordeaux Population Health Research Center, Team LEHA, UMR 1219, F-33000 Bordeaux, France; Cecile.Delcourt@isped.u-bordeaux2.fr; 13 Hôpital Intercommunal de Créteil, University Paris Est, 94010 Créteil, France; eric.souied@chicreteil.fr; 14 Department of Ophthalmology, Tufts University School of Medicine, Boston, MA 02111, USA; jseddon@tuftsmedicalcenter.org; 15 Ophthalmic Epidemiology and Genetics Service, Tufts Medical Center, Boston, MA 02111, USA

**Keywords:** vitamin D, age-related macular degeneration, inflammation, angiogenesis

## Abstract

In recent years, the relationship between vitamin D and health has received growing attention from the scientific and medical communities. Vitamin D deficiencies have been repeatedly associated with various acute and chronic diseases, including age-related macular degeneration (AMD). Its active metabolite, 1α,25-dihydoxy vitamin D, acts as a modulator of cell proliferation, differentiation and apoptosis, and cumulative data from experimental and observational studies suggest that relatively a lower vitamin D status could be a potential risk factor for the development of early and/or late AMD. Herein, we made a narrative review of the mechanisms linking a potential role of vitamin D with the current concepts of AMD pathophysiology.

## 1. Introduction

Age-related macular degeneration (AMD) is a chronic, progressive degenerative disease affecting the macula and reducing central visual acuity in advanced stages. This is the leading cause of irreversible visual impairment in the elderly population in developed countries, accounting for 7% of all blindness worldwide [1,2]. The prevalence of AMD is increasing, primarily due to increased life expectancy [3]. The exact pathophysiology is only partly understood [4,5]. However, our knowledge of the disease and its underlying mechanisms have progressed since the last decade. The pathogenesis of AMD is the result of complex multifactorial interactions between metabolic, functional, genetic, and environmental factors [5]. Advancing age acts as the strongest predictor, and AMD is more frequently found in Caucasians than African Americans [6]. There is also an increased risk in individuals with positive family histories [7,8]. Smoking is the most important modifiable risk factor, followed by lifestyle, diet and nutrition [5].

Oxidation, inflammation and angiogenesis in the retinal pigment epithelium (RPE) and choriocapillaries are thought to play central roles in AMD pathogenesis, leading to dysfunction of the RPE, Bruch’s membrane and choriocapillaries, and progressively leading to photoreceptor loss [9,10]. The current clinical classification is based on fundus lesions assessed within two disc diameters of the fovea in persons older than 55 years [11]. Early stages of AMD are usually asymptomatic and are clinically characterized by the accumulation of drusen of medium size (between 63 and 125 μm), with pigmentary abnormalities [11]. Intermediate AMD is characterized by larger drusen (>125 μm) and/or pigmentary abnormalities) and may progress to advanced (or late) forms, i.e., atrophic or exudative/neovascular AMD. In atrophic AMD (“geographic atrophy”), there is a progressive loss of RPE cells and corresponding photoreceptor cells. The neovascular form of AMD is characterized by abnormal proliferation of choroidal capillaries, which may subsequently cause accumulation of intra- and sub-retinal and sub-RPE fluid as well as hemorrhages. Progression is typically faster than in atrophic AMD and may lead, if left untreated, to severe and permanent visual impairment.

There is currently no therapy for atrophic AMD, although intravitreal injection of anti-vascular endothelial growth factor (VEGF) agents may slow or halt the progression of exudative AMD [12]. Primary or secondary prevention appears mandatory in order to limit the burden of the disease [3,5]. Lifestyle modifications (e.g., smoking cessation, weight loss) and a healthy diet have been recommended during all stages of AMD [5,13]. Although there is no good evidence that oral vitamin and mineral supplementation may prevent AMD development in the general population [14], supplementation with antioxidants (vitamin C, vitamin E, lutein, and zeaxantine) may slow disease progression to certain disease stages [15,16,17]. Other nutrients, including omega-3 fatty acids or resveratrol, have plausible biological protective effects and are under investigation to reduce the risk of AMD [15]. Furthermore, recent data from epidemiological and experimental studies point towards a potential role of vitamin D in AMD pathophysiology [18,19]. Our objective in this paper was to review the mechanisms linking the role of vitamin D with current concepts of AMD pathophysiology since the first publication of the observational study suggested an inverse association between vitamin D status and the risk of early AMD [20].

## 2. Literature Review Method

Our narrative review was based on a Medline search and the selection of the most relevant publications between January 2007 and December 2016 with the search terms “age-related macular degeneration”, “vitamin D”, “inflammation”, “oxidation”, and “angiogenesis”. We selected all experimental, genetic and epidemiologic studies, supporting or not, a link between vitamin D and AMD.

## 3. Vitamin D Function and Health

### 3.1. Source, Metabolism, and Storage

**Figure 1:**
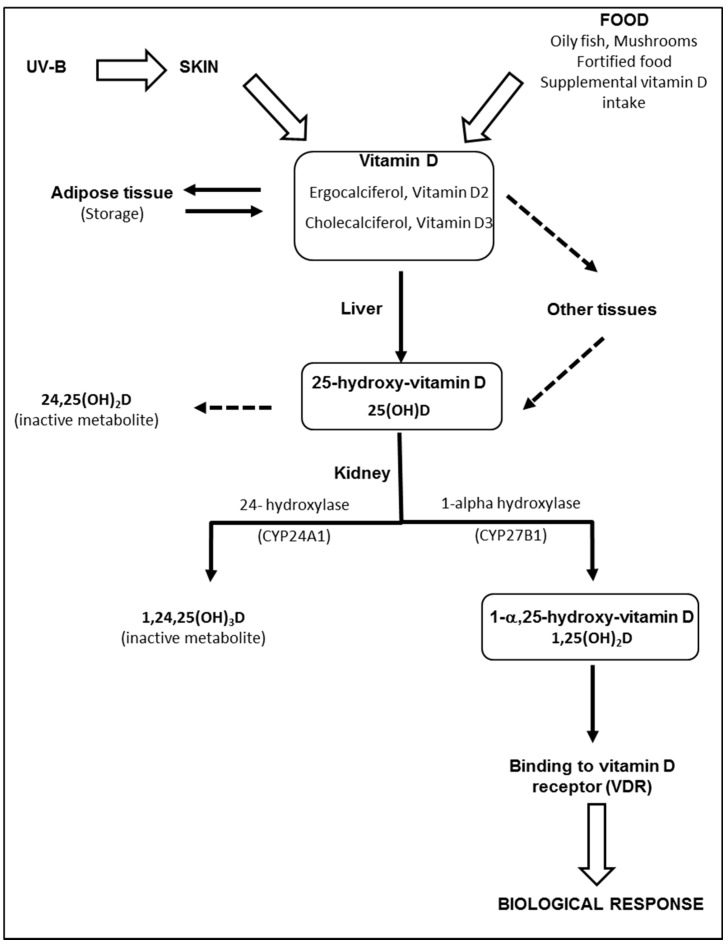
Metabolism of vitamin D. Vitamin D (ergocalciferol and/or cholecalciferol) is produced and excreted by basal skin keratinocytes exposed to ultraviolet radiation (UV-B), or directly provided by food. While skin vitamin D is transported into the liver bound to binding proteins (DBP), dietary vitamin D is absorbed by the gastro-intestinal tract and transported to the liver via the venous circulation and chylomicron remnants. Part of the vitamin D produced is stored in fat cells and may serve as an endogenous source of vitamin D. In the liver, vitamin D2 and vitamin D3 are hydroxylated in position 25 by several enzymes found in microsomal or mitochondrial fractions. Once produced in the liver, 25(OH)D is released into the bloodstream whilst bound to DBP. Alternatively, vitamin D can be metabolized in 25(OH)D in other tissues. In the kidney, 25(OH)D is converted to the active metabolite, 1,25(OH)_2_D, through the action of the enzyme 1-alpha-hydroxylase (CYP27B1), located in the proximal tubules. In excess, 1,25(OH)_2_D and 25(OH)D activate 24-hydroxylase (CYP24A1) and are degraded into 24-hydroxylated products, i.e., 24,25(OH)_2_D and 1,24,25(OH)_3_D, which have no biological activity. Once produced in the kidney, 1,25(OH)_2_D is released and transported into the bloodstream and is mainly bound to DBP until it reaches target tissues expressing the vitamin D receptor.

The term “vitamin D” is used collectively to identify two molecules that differ chemically in their side chains: vitamin D2 (ergocalciferol), derived from ergosterol in irradiated plants and vitamin D3 (cholecalciferol), found in fish oils, eggs, and animal fats. Most vitamin D is actually produced by skin following sunlight exposure [21]. Vitamin D3 is produced and excreted by basal skin keratinocytes exposed to ultraviolet radiation (UV-B), leading to the photolysis of 7-dehydrocholesterol (7-DHC) to pre-vitamin D3. Excess pre-vitamin D3 is converted into various inactive metabolites [22]. Once produced by the skin, vitamin D binds to a specific binding protein (DBP) and is released into the bloodstream. Part of the vitamin D produced is stored in fat cells [23]. Vitamin D, produced by the skin or supplied by food, is biologically inert and requires two subsequent hydroxylation processes in the liver and kidneys to produce active metabolites, as described in Figure 1. The liver is generally considered the primary, and likely the sole source, of 25(OH)D production. However, other enzymes have 25-hydroxylase activity and may potentially affect levels of 25(OH)D in the blood and possibly other tissues [24]. Contrary to the liver, 25-hydroxylases, both renal enzymes—CYP27B1 and CYP24A1—are tightly controlled [24]. Negative feed-back is produced by 1,25(OH)_2_D, through down-regulation of CYP27B1 and up-regulation of CYP24A1. The half-life of 1,25(OH)_2_D in plasma is relatively short (only several hours) [25], compared to the half-life of 25(OH)D (about 3 weeks). Therefore, serum 25(OH)D is considered the best biomarker of vitamin D status [25]. Proximal tubular epithelial (renal) cells are not the only source of 1,25(OH)_2_D production. CYP27B1 is also expressed in a number of extrarenal sites, including the gastrointestinal tract, skin, vasculature, placenta and immune cells [26]. Although the physiological impact of extrarenal CYP27A1 is still controversial [26], local synthesis of 1,25(OH)_2_D provides the basis for a paracrine or autocrine function.

### 3.2. Mode of Action

Vitamin D is a secosteroid that is structurally analogous to the steroid hormones (e.g., estradiol, cortisol, and aldosterone), but with an open B-ring. Similar to other steroid hormones, vitamin D functions according to two modes of action: a mechanism mediating gene transcription (genomic action) and a rapid non-transcriptional action, mediated by the activation of secondary messengers and phosphokinase activation (non-genomic action) [27,28]. The genomic pathway is mediated by the binding of 1,25(OH)_2_D with a high affinity vitamin D receptor (VDR). When activated, the VDR acts as a transcriptional factor [24,27] and may directly or indirectly control 200 to 2000 genes in various tissues and cells [29]. This includes genes involved in mineral and bone homeostasis, but also genes controlling cell proliferation, differentiation, and apoptosis [27,29,30]. The VDR is ubiquitously expressed throughout the human body [31], including in immune cells, endothelial cells and vascular smooth muscle cells [32], but also in eye tissues, including the retina [33]. It was recently demonstrated that vitamin D3 supplementation (400 to 2000 IU/day for 8 weeks) is associated with related alterations of 291 genes, including 17 genes known to play important roles in transcriptional regulation, immune function, apoptosis, and responses to stress [34]. The non-genomic pathway involves the interaction of 1,25(OH)_2_D with a specific receptor localized to the plasma membrane of target cells [28]. Based on cell type, signal transduction may involve different secondary messengers and cytosolic kinase systems, leading to biological effects that include the regulation of cell proliferation, differentiation, or apoptosis [30].

### 3.3. Association between Vitamin D and Health Outcomes

**Figure 2:**
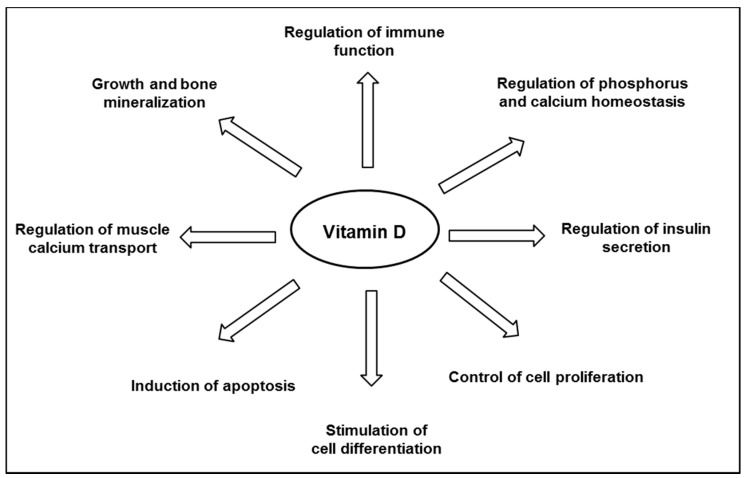
Major biological functions of vitamin D.

Vitamin D is well known for its major role in bone mineral homeostasis; it promotes the transport of calcium and phosphate, ensuring adequate bone mineralization [35]. Vitamin D is thought to have other biological functions (Figure 2), and observational studies have suggested an inverse relationship between the plasma level of 25(OH)D and the risk of developing various chronic diseases, including cancers, infections, cardiovascular diseases, auto-immune diseases, and diabetes [22,24]. Although a link is supported by experiments in vitro or animal studies, as reviewed by others [32,36,37,38], not all literature supports a protective association between vitamin D and chronic disease outcomes. There has still no obvious causality link shown in interventional studies, and consistent data from randomized clinical trials are still scarce, as reviewed by Bikle [24]. Thus, to date, the Scientific Advisory Committee on Nutrition (SACN) has recognized the critical role of vitamin D in bone health, but not in other chronic diseases [39].

## 4. The Links between Vitamin D and AMD

### 4.1. Vitamin D and AMD Pathophysiology

**Figure 3:**
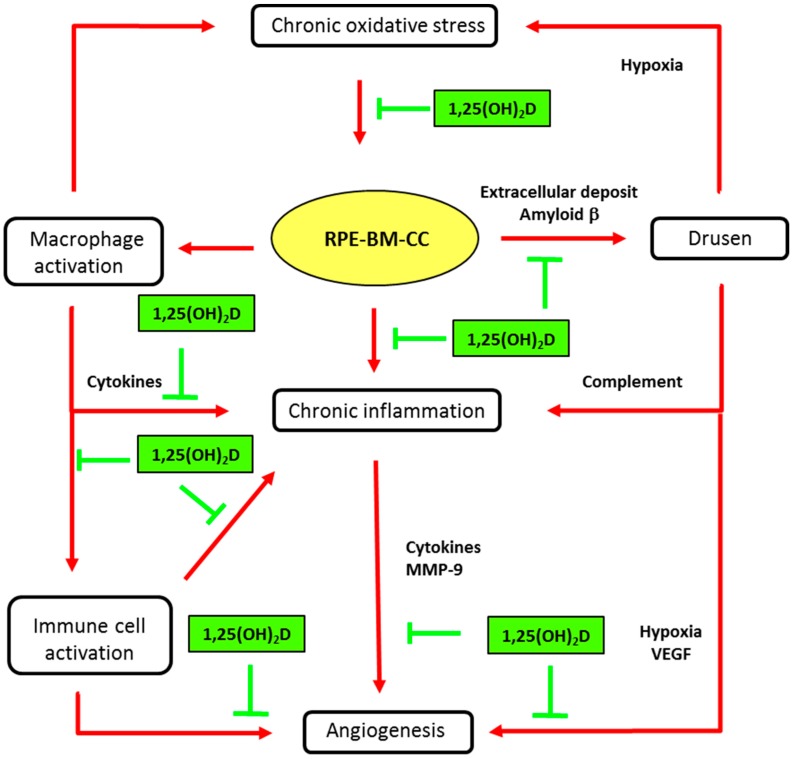
Main biological mechanisms in age-related macular degeneration (AMD) and putative vitamin D effects. Inhibitory activities of vitamin D (1,25(OH)_2_D) are indicated in green truncated arrows and mechanisms of AMD pathophysiology in red arrows. AMD is characterized by progressive degeneration of the macula, involving the retinal pigment epithelium (RPE), the Bruch’s membrane (BM), and alterations in choroidal capillaries (CC). Chronic oxidative stress in senescent RPE is a key event in maintaining macular damage and initiating early AMD. The release of cell debris and the accumulation of specific deposits (drusen) is the hallmark histopathological feature of eyes with early and intermediate AMD. Vitamin D may prevent the risk for developing early and intermediate AMD, by inhibiting oxidative stress, inhibiting extracellular amyloid deposits and inhibiting macrophage activation. Advanced dry AMD is characterized by atrophy of RPE cells and choriocapillaries. RPE dysregulation, due to oxidative stress and inflammatory reactions, may lead to abnormal angiogenesis, leading to neovascular AMD. Vitamin D may reduce the risk or slow the development of neovascular AMD by inhibiting angiogenesis or immune cell activation (see text for details).

A possible physiological role of vitamin D in the retina is supported by evidence that the vitamin D receptor (VDR) and the enzymes involved in the metabolism of vitamin D (CYP27B1 and CYP24A1) are expressed in the retina, RPE and choroid. The presence of VDR was demonstrated through immunohistochemical staining in the inner and outer segments of retinal photoreceptors and the RPE [33]. The presence of the VDR gene in resting retinal and choroidal endothelial cells was then confirmed by molecular biology [40,41]. Interestingly, Morrison et al. [40] reported that VDR expression in RPE/choroid tissues was generally observed at or below the level of detection, but was at significantly higher levels in several donors, concomitant with an increased expression of genes involved in local inflammatory responses. More recently, it was shown that the human RPE cell line, ARPE-19, was able to convert 25(OH)D to 1,25(OH)_2_D [42]. The presence of the VDR and enzyme 1-alpha-hydroxylase (CYP27B1) in retinal and RPE/choroidal cells strongly suggests that vitamin D may function in a paracrine/autocrine manner. Thus, a major limiting step in vitamin D function may be the bioavailability of 25(OH)D, as lower systemic levels may directly diminish the amount of active vitamin D in the macula [42]. Protective effects of vitamin D, which prevent the development of early and/or late AMD, have been proposed, based on experimental in vitro and in vivo studies, as reviewed by Wang & Hartnett [4] and Parmeggiani & Romano [43]. These include pathogenic steps centered on oxidation, inflammation and angiogenesis, as illustrated in Figure 3.

#### 4.1.1. Inhibition of Chronic Oxidative Stress

One major physiological role of RPE cells is phagocytosis and intracellular degradation of shed photoreceptor outer segments [44]. Intracellular degradation is mediated by the generation of free reactive oxygen species (ROS) and lysosomal enzymes. Cellular debris is normally digested by the RPE cells. However, in senescent RPE cells, oxidative stress is increased, leading to cell damage, RPE dysfunction and release of abnormal extracellular matrix deposits. Vitamin D has been shown to be protective against oxidative stress in various cell lines and animal models [45,46]. Recently, in a mouse cone cell line (661W), 1,25(OH)_2_D was shown to decrease the generation of ROS in H_2_O_2_-stimulated cells, by modulating the expression of antioxidant enzymes (catalase, superoxide dismutase, and glutathione peroxidase) [18].

#### 4.1.2. Inhibition of Amyloid Beta Protein Deposits

A pathogenic role for amyloid beta protein (Aβ) in AMD was proposed in studies showing that Aβ increases VEGF expression in human RPE cells [47]. A large amount of Aβ is released by senescent RPE cells [48] and Aβ is a major component of drusen [49]. In addition, Aβ is considered a primary activator of the complement cascade and inflammation, as reviewed by Akiyama et al. [50]. The deleterious role of Aβ in the pathophysiology of AMD has been suggested by animal studies, which have shown that intraperitoneal administration of anti-Aβ antibodies in aged mice leads to a reduction of Aβ deposition in the retina and improves electroretinogram (ERG) deficits [51]. On the other hand, vitamin D has been recently associated with the clearance of Aβ deposits and the improvement of retinal function in aged mice treated with subcutaneous injections of vitamin D [52]. The proposed mechanism involves the activation of macrophage phagocytosis of Aβ deposits by 1,25(OH)_2_D, and their removal from the Bruch’s membrane. These results may indicate a role of vitamin D supplementation in prevention or treatment of early AMD.

#### 4.1.3. Inhibition of Chronic Inflammation

AMD can be considered a low-grade chronic inflammatory disease, where immunocompetent cells, such as macrophages and lymphocytes, are present in the chorioretinal tissues affected by AMD (for review see Parmeggiani et al. [43]). In animal experiments, inflammatory cells are located in the RPE, Bruch’s membrane, choroid, and outer and inner segments, and were more abundant in areas with increasing damage [53]. In the aging retina, two inflammatory pathways are affected as a result of age-related tissue stress: the complement cascade (for review see Khandadia et al. [54]) and the tissue resident macrophage (retinal microglia) activation pathway [55]. Inflammatory events ultimately lead to protein damage, aggregation and degeneration of RPE and are therefore key elements in the pathogenesis of AMD [3]. On the other hand, there are several lines of evidence consistent with an anti-inflammatory role of vitamin D in the pathogenesis of AMD. Vitamin D was shown to inhibit immune reactions in chronic inflammatory diseases, as reviewed by Hewison et al. [56]. Cells of the myeloid lineage are known to produce CYP27B1 hydroxylase, leading to local production of 1,25(OH)_2_D. Activated macrophages express the VDR and are thus vitamin D responsive cells. One major effect of vitamin D on activated macrophages is potent suppression of pro-inflammatory events mediated by interferon-gamma (INF-γ) [57]. This is consistent with the study in mice, which showed that vitamin D administration reduces the number of activated macrophages and promotes a shift from pro-inflammatory cytokine-secreting macrophages to an amoeboid cell type, still capable of phagocytosis [52]. In the RPE/choroid, the inhibition of the inflammatory reaction mediated by cytokines, which are secreted by macrophages/microglia (Interleukin(IL)-1, IL-6 and tumor necrosis factor -1 (TNFα)), may prevent RPE degeneration and apoptosis [58,59].

Adaptive immunity in AMD is a recent investigational field, and data suggests that dysregulation of immune responses could contribute to different processes associated with AMD, such as RPE atrophy, neovascularization, and retinal degeneration [60]. It was reported that the C5a complement component induced IL-22 and IL-17 expression by human peripheral CD4+ T cells, and that elevated levels of these cytokines were present in AMD patients [61]. This provides a potential link between innate and adaptive immunities in the pathogenesis of AMD. Vitamin D, as a potential inhibitor of immune cell recruitment, proliferation and activation, may protect against the development of AMD. Indeed, vitamin D metabolizing enzymes and vitamin D receptors are present in many immune cells, including antigen-presenting cells, T cells, B cells and monocytes [38,56]. Vitamin D (1,25(OH)_2_D) has been shown to suppress proinflammatory cytokines, in part by altering T-cell function toward a Th2 (anti-inflammatory) rather than a Th1 (pro-inflammatory) response [56]. As reviewed by Prietl et al. [38], treatment of T cells with 1,25(OH)_2_D or its analogs, inhibits the secretion of proinflammatory Th1 (IL-2, INFγ, TNFα), Th9 (IL-9) and Th22 (IL-22) cytokines, but promotes the production of more anti-inflammatory Th2 cytokines (IL-3, IL-4, IL-5, IL-10). The production of IL-17 produced by Th17 cells is also inhibited by vitamin D [62].

#### 4.1.4. Vitamin D and Angiogenesis in AMD

It is well established that angiogenesis plays a major role in the development and progression of AMD. VEGF, the most potent inducer of endothelial activation, is a modulator of vascular permeability in the macula. The RPE is capable of secreting a variety of growth factors, including VEGF, in order to maintain the physiological angiogenesis necessary for physiological development of the choroidal vasculature [63]. RPE dysregulation due to oxidative stress and inflammatory reactions may lead to abnormal angiogenesis [64]. On the other hand, hypoxia, caused by drusen accumulation in the Bruch’s membrane, is considered a strong activator of VEGF secretion by RPE cells [65]. Inflammatory cytokines, such as TNFα released by activated microglia and IL-17 produced by Th17 cells, may also induce angiogenesis in choroidal endothelial cells [4]. As demonstrated in tumor cells, vitamin D is a potent inhibitor of angiogenesis due to its effects on endothelial cells and interruption of the angiogenesis signaling pathway [66]. In various human cancer cells, it has been shown that the anti-angiogenic activity of 1,25(OH)_2_D is mediated by inhibition of the transcription of hypoxia-inducible factor (HIF-1), which is a well-known inducer of VEGF under conditions of hypoxia [67]. A similar regulatory role for HIF-1 was shown in the pathogenesis of AMD [68]. In vitro and in vivo experiments, performed by Mantel et al., showed that 1,25(OH)_2_D inhibits specific stages of the angiogenic process in a dose-dependent manner [69]. Vitamin D (1,25(OH)_2_D) inhibits VEGF-induced endothelial cell sprouting and elongation and also has a small, but significant, inhibitory effect on VEGF-induced endothelial cell proliferation. In experiments carried out by Albert et al. using a mouse model of oxygen-induced ischemic retinopathy, animals treated with 1,25(OH)_2_D had significant decreases in retinal neovascularization relative to control animals, although the levels of ocular VEGF were similar in treated and control animals [70]. A possible mechanism may include induction of endothelial cell apoptosis [69,70]. On the other hand, inhibition of the production of the metalloproteinase, MMP-9, released by inflammatory cells may be another pathway controlled by vitamin D. In TNFα-stimulated keratinocytes, vitamin D was shown to attenuate the production of the metalloproteinase, MMP-9 [71], which is suspected to play a role in choroidal neovascularization, by altering the Bruch’s membrane [72,73].

### 4.2. Vitamin D Status and AMD

#### 4.2.1. Association between Vitamin D Plasma Levels and AMD

The association between vitamin D and AMD is a relatively new investigational field. Observational studies conducted over the past decade, to investigate the relationship between vitamin D status and AMD risk, have led to controversial results. A recent meta-analysis, of 11 observational studies [74] indicated that low circulating levels of 25(OH)D (<50 nmol/L (20 ng/mL)) were significantly associated with late AMD, with an odds ratio (OR) of 2.18 (95% CI: 1.34–3.56). However, the association was not statistically significant in early AMD. In another recent review, with slightly different study criteria, Wu et al. concluded that there was no evidence to indicate an inverse association between blood vitamin D levels and risk of any stages or subphenotypes of AMD [75].

Although such meta-analyses overcome some statistical issues, the selected studies showed high heterogeneity in design, including population representativeness, ethnicity, time and methods to determine vitamin D status as well as AMD classification. In their meta-analysis, Wu et al. reviewed the quality of individual observational studies, based on 10 methodologic criteria that are not always fulfilled [75]. There are several well conducted observational studies which have shown an inverse relationship between vitamin D plasma levels and early AMD [20,76], or late atrophic and/or neovascular AMD [19,77]. However, other studies, with lower quality scores, have not shown significant associations between vitamin D status and prevalent AMD [78,79,80].

Finally, all but one of the published epidemiological studies (in particular those included in the two meta-analyses) were cross-sectional or case-control studies, with 25(OH)D being measured after the diagnosis of AMD. Although cross-sectional studies are useful for exploring a possible association between AMD and vitamin D, they are not designed to determine causality. As recognized by others, the reduced vitamin D levels observed in cross-sectional analyses of patients with AMD may be related to visual impairment, more sedentary indoor activities, less exposure to sunlight and therefore, less vitamin D skin production [19]. For this reason, prospective studies are preferable and avoid issues related to assessment of vitamin D levels after the development of advanced disease.

#### 4.2.2. Relationship between Dietary Vitamin D and AMD

To date, interventional studies assessing the effect of vitamin D supplementation in preventing the onset or progression of AMD are lacking. Thus, there are no specific dietary recommendations regarding vitamin D for primary or secondary prevention of AMD, despite the high prevalence of vitamin D deficiency or insufficiency in the general population [81,82]. However, the relationship between dietary vitamin D and AMD has been investigated in several studies. Parekh et al. found that milk intake (fortified in vitamin D in the USA) was inversely associated with early AMD (OR: 0.75; 95% CI, 0.6–0.9) and fish intake was inversely associated with advanced AMD (OR: 0.41; 95% CI, 0.2–0.9) [20]. In Caucasian male monozygotic twin pairs with discordant AMD phenotypes, Seddon et al. reported that higher dietary intakes of vitamin D (assessed using a food frequency questionnaire) were present in twins with less severe AMD (*p* = 0.01) and smaller drusen sizes (*p* = 0.05) relative to co-twins—adjusted for smoking and age [83]—providing evidence that vitamin D could be involved in the etiology of AMD. In a recent Japanese case-control study, patients with neovascular AMD and control subjects, randomly selected from the population, aged ≥65 years, were assessed, using a brief-type self-administered questionnaire (BDHQ) on diet history. Logistic regression analyses, adjusted for smoking, age, sex, chronic diseases, supplement use, and alcohol consumption, demonstrated that a low intake of vitamin D, together with other nutrients, including n-3 fatty acid, alpha-tocopherol, zinc, vitamin C, and beta-carotene, was significantly (*p* < 0.001) associated with neovascular AMD [84].

#### 4.2.3. Genetic Link between Vitamin D and AMD

Recent insight into pathophysiologic triggers has indicated that AMD involves the interaction of multiple genetic and environmental factors [5,12,85]. The interrelationship between gene polymorphisms and vitamin D has been suggested in several studies, providing additional links between vitamin D and AMD pathophysiology. A study performed in extremely discordant siblings (i.e., one sibling had the neovascular form of AMD while the discordant sibling had no sign of AMD and was older than 65 years) suggested that a single point variant in CYP24A1 (the gene encoding the catabolizing enzyme of the vitamin D pathway) influenced AMD risk, although this association has not been replicated [40]. Several gene polymorphisms in CYP2Rl (the 25-hydroxylase which converts vitamin D into 25(OH)D) and in VDR were also suggested to increase risk [86]. Moreover, an interaction between vitamin D status and single nucleotide polymorphisms (SNPs) in the complement factor H (*CFH* Y402H) and complement factor I (*CFI*) genes has been suggested [86]. Overall, compared to subjects with adequate blood 25(OH)D levels (i.e., ≥30 ng/mL), there was a 2.6-fold (95% CI: 1.3–5.2) increased likelihood of AMD in 25(OH)D deficient subjects (i.e., <12 ng/mL), a 3.4-fold (95% CI: 1.1–10.9) increased likelihood in subjects carrying one risk allele for *CFH* Y402H and a 6.7-fold (95% CI: 1.6–28.2) increased likelihood in subjects carrying two risk alleles. Similar trends were found with a *CFI* SNP. This is noteworthy, since these two proteins—*CFH* and *CFI*—work in interconnection to inhibit the complement cascade, which plays a central role in drusen formation in AMD [3,43]. Therefore, in the case of vitamin D deficiency, the ability to suppress the local inflammatory response may be impaired, leading to an increased risk of AMD. Another gene product, *HTRA1*, a serine protease which was shown to be expressed in the drusen of AMD patients [87], may be upregulated by vitamin D. A gene polymorphism in a vitamin D-responsive element in the promoter region of the *HTRA1* gene may suppress this effect [88]. These possible associations support a link between vitamin D and genetic susceptibility to AMD.

## 5. Conclusions and Future Perspectives

Vitamin D can be considered a steroid hormone which binds to high affinity receptors. Experimental studies have suggested that vitamin D can control the expression of genes involved in oxidative stress, inflammation, and angiogenesis. In the macula, vitamin D may preserve the function of the retinal pigmentary epithelium and choroidal cells, through a paracrine/autocrine pathway. It is thus possible that the bioavailability of 25(OH)D circulating in blood is a limiting step in the protective effect of vitamin D. On the other hand, observational studies, including population-based studies, suggest an association between vitamin D deficiency and a higher risk of early and/or late AMD. This is consistent for a role of vitamin D in the pathophysiology of AMD. The potential causal association between vitamin D and AMD encourages future clinical research. In the interim, due to insufficient data, there is still no recommendation to screen for vitamin D deficiencies in patients at risk of AMD. However, all individuals may benefit by increasing their levels of vitamin D, through all possible means, including sun exposure, dietary recommendations, vitamin D-enriched foods, and vitamin D supplementation.
